# Tracking Social Media Discourse About the COVID-19 Pandemic: Development of a Public Coronavirus Twitter Data Set

**DOI:** 10.2196/19273

**Published:** 2020-05-29

**Authors:** Emily Chen, Kristina Lerman, Emilio Ferrara

**Affiliations:** 1 Information Sciences Institute University of Southern California Marina del Rey, CA United States

**Keywords:** COVID-19, SARS-CoV-2, social media, network analysis, computational social sciences

## Abstract

**Background:**

At the time of this writing, the coronavirus disease (COVID-19) pandemic outbreak has already put tremendous strain on many countries' citizens, resources, and economies around the world. Social distancing measures, travel bans, self-quarantines, and business closures are changing the very fabric of societies worldwide. With people forced out of public spaces, much of the conversation about these phenomena now occurs online on social media platforms like Twitter.

**Objective:**

In this paper, we describe a multilingual COVID-19 Twitter data set that we are making available to the research community via our COVID-19-TweetIDs GitHub repository.

**Methods:**

We started this ongoing data collection on January 28, 2020, leveraging Twitter’s streaming application programming interface (API) and Tweepy to follow certain keywords and accounts that were trending at the time data collection began. We used Twitter’s search API to query for past tweets, resulting in the earliest tweets in our collection dating back to January 21, 2020.

**Results:**

Since the inception of our collection, we have actively maintained and updated our GitHub repository on a weekly basis. We have published over 123 million tweets, with over 60% of the tweets in English. This paper also presents basic statistics that show that Twitter activity responds and reacts to COVID-19-related events.

**Conclusions:**

It is our hope that our contribution will enable the study of online conversation dynamics in the context of a planetary-scale epidemic outbreak of unprecedented proportions and implications. This data set could also help track COVID-19-related misinformation and unverified rumors or enable the understanding of fear and panic—and undoubtedly more.

## Introduction

The first cases of coronavirus disease (officially named COVID-19 by the World Health Organization [WHO] on February 11, 2020) were reported in Wuhan, China, in late December 2019; the first fatalities were reported in early 2020 [1]. The fast-rising infections and death toll led the Chinese government to quarantine the city of Wuhan on January 23, 2020 [1]. During this period, other countries began reporting their first confirmed cases of the disease, and on January 30, 2020, the WHO announced a Public Health Emergency of International Concern. With more countries reporting cases of the disease, and infections rapidly escalating in some regions of the world, including South Korea, Iran, and Italy, the WHO declared COVID-19 a pandemic [2]. At the time of this writing, COVID-19 has been reported in 185 countries, leaving governments all over the world scrambling for ways to contain the disease and lessen its adverse consequences to their people's health and economy [3].

Preventative measures implemented by national, state, and local governments now affect the daily routines of millions of people worldwide [4]. *Social distancing*, the most widely used of such measures, aims to curtail new infections by reducing physical contact between people [5]. Social distancing measures have led to the cancellation of sporting events and conferences [6], closures of schools and colleges [7], and has forced many businesses to require their employees to work from home [8]. As more and more social interactions move online, the conversation around COVID-19 has continued to expand, with growing numbers turning to social media for both information and company [9,10]. Platforms such as Twitter have become central to the technological and social infrastructure that allows us to stay connected even during crises.

We describe a Twitter data set about COVID-19-related online conversations that we are sharing with the research community. People all over the world take to Twitter to express opinions and engage in dialogue in a public forum, and, with Twitter’s open application programming interface (API), has proven to be an invaluable resource for studying a wide range of topics. Twitter has long been used by the research community as a means to understand dynamics observable in online social networks, from information dissemination [11,12] to the prevalence and influence of bots and misinformation [13,14]. More importantly during the current COVID-19 pandemic, Twitter provides researchers the ability to study the role social media plays in the global health crisis [15-19]. We hope that this data will spur new research about the social dimensions of the pandemic.

We began collecting data in real time from Twitter, with the earliest tweets dating to January 21, 2020, by tracking COVID-19-related keywords and accounts. Here, we describe the data collection methods, document initial data statistics, and provide information about how to obtain and use the data.

## Methods

### Overview

We have been actively collecting tweets since January 28, 2020, leveraging Twitter's streaming API [20] and Tweepy [21] to follow specific keywords and accounts that were trending at the time. When we started collecting tweets, we also used Twitter's search API [22] on the same keywords to gather related historical tweets. Thus, the earliest tweets in our collection date back to January 21, 2020. Since then, we have incrementally added keywords and accounts to follow based on the conversations occurring on Twitter at any time. We have collected over 72 million tweets from inception to March 21, 2020, constituting roughly 600 GB of raw data, and are still collecting data to this day.

Our collection relies upon publicly available data and is hence registered as IRB (institutional review board) exempt by the University of Southern California IRB (approved protocol UP-17-00610). We release the data set with the stipulation that those who use it must comply with Twitter’s Terms and Conditions [23].

### Tracked Keywords and Accounts

By continuously monitoring Twitter's trending topics, keywords, and sources associated with COVID-19, we did our best to capture conversations related to the outbreak.

Twitter's streaming API returns any tweet containing the keyword(s) in the text of the tweet, as well as in its metadata; therefore, it is not always necessary to have each permutation of a specific keyword in the tracking list. For example, the keyword “Covid” will return tweets that contain both “Covid19” and “Covid-19.” We list a subset of the keywords and accounts that we are following in Tables 1 and 2, respectively, along with the date we began tracking them. There are some keywords that overlap due to an included keyword being a substring of another, but we included both for good measure. The keyword choices in the current data set are all in English, so there is a heavy bias toward English tweets and events related to English-speaking countries. Due to the evolving nature of the pandemic and online conversations, these tables will expand as we continue to monitor Twitter for additional keywords and accounts to add to our tracking list.

**Table 1:** A sample of the keywords that we are actively tracking in our Twitter collection; see the GitHub repository for a full list of all tracked keywords (v1.8—May 8, 2020) [24].

Tracked since	Keyword
1/21/2020	Coronavirus; Corona; CDC; Ncov; Wuhan; Outbreak; China
1/22/2020	Koronavirus; Wuhancoronavirus; Wuhanlockdown; N95; Kungflu; Epidemic; Sinophobia
2/16/2020	Covid-19
3/2/2020	Corona virus
3/6/2020	Covid19; Sars-cov-2
3/8/2020	COVID–19
3/12/2020	COVD; Pandemic
3/13/2020	Coronapocalypse; CancelEverything; Coronials; SocialDistancing
3/14/2020	Panic buying; DuringMy14DayQuarantine; Panic shopping; InMyQuarantineSurvivalKit
3/16/2020	chinese virus; stayhomechallenge; DontBeASpreader; lockdown
3/18/2020	shelteringinplace; staysafestayhome; trumppandemic; flatten the curve
3/19/2020	PPEshortage; saferathome; stayathome
3/21/2020	GetMePPE
3/26/2020	covidiot
3/28/2020	epitwitter
3/31/2020	Pandemie

**Table 2:** Account names that we are actively tracking in our Twitter collection (v1.8—May 8, 2020).

Tracked since	Account name
1/22/2020	PneumoniaWuhan; CoronaVirusInfo; V2019N; CDCemergency; CDCgov; WHO; HHSGov; NIAIDNews
3/15/2020	DrTedros

## Results

### Releases

Our data collection will continue uninterrupted for the foreseeable future. As the pandemic continues to run its course, we anticipate that the amount of data will grow significantly. The data set is available on GitHub [24] and is released in compliance with the Twitter's Terms and Conditions, under which we are unable to publicly release the text of the collected tweets. We are, therefore, releasing the Tweet IDs, which are unique identifiers tied to specific tweets. The Tweet IDs can be used by researchers to query Twitter’s API and obtain the complete tweet object, including tweet content (text, URLs, hashtags, etc) and authors’ metadata. This process to retrieve the full tweet object from Twitter starting from a Tweet ID is referred to as *hydration*. There are several easy-to-use tools that have been developed for such purposes, including the *Hydrator* [25] and *Twarc* [26], but one could also directly use Twitter’s API to retrieve the desired data. This data set can also be found on Harvard Dataverse [27]. Table 3 displays basic statistics, including collection period and number of tweets in that respective release, for all current releases (as of May 15, 2020).

There are a few known gaps in the data, which are listed in Table 4. Due to Twitter API restrictions on free data access, we were unable to recover data from the listed times, as Twitter only provides free access to tweets returned from their streaming API from the past week. To request access, interested researchers will need to agree upon the terms of usage dictated by the chosen license.

All of the Tweet ID files are stored in folders that indicate the year and month the tweet was posted (YEAR-MONTH). The individual Tweet ID files each contain a collection of Tweet IDs, with the file names all beginning with the prefix “coronavirus-tweet-id-” followed by the year, month, date, and hour the tweet was posted (YEAR-MONTH-DATE-HOUR).

We note that if a tweet has been removed from the platform, researchers will not be able to obtain the original Tweet.

**Table 3:** List of all releases and their statistics.

Release version	Release date	Data collection period	Tweets, n
v1.0	3/17/2020	3/05/2020 - 3/12/2020	8,919,411
v1.1	3/23/2020	1/21/2020 - 3/12/2020	63,616,072
v1.2	3/31/2020	1/21/2020 - 3/21/2020	72,403,796
v1.3	4/11/2020	1/21/2020 - 4/03/2020	87,209,465
v1.4	4/13/2020	1/21/2020 - 4/10/2020	94,671,486
v1.5	4/20/2020	1/21/2020 - 4/17/2020	101,771,227
v1.6	4/26/2020	1/21/2020 - 4/24/2020	109,013,655
v1.7	5/04/2020	1/21/2020 - 5/01/2020	115,929,358
v1.8	5/11/2020	1/21/2020 - 5/08/2020	123,113,914

**Table 4:** Known gaps in the data set in UTC (v1.8—May 8, 2020).

Date	Time
2/1/2020	4:00 - 9:00 UTC
2/8/2020	6:00 - 7:00 UTC
2/22/2020	21:00 - 24:00 UTC
2/23/2020	0:00 - 24:00 UTC
2/24/2020	0:00 - 4:00 UTC
2/25/2020	0:00 - 3:00 UTC
3/2/2020	Intermittent internet connectivity issues

#### The Most Recent Release (Release v1.8—May 11, 2020)

Our 9th release spans January 21, 2020, through May 8, 2020. The data set available now contains tweets from January 21, 2020 (22:00 UTC), through May 8, 2020 (21:00 UTC), with 123,113,914 tweets. The language breakdown of the tweets can be found in Table 5. A subset of the keywords and accounts that were followed during this timeframe can be identified by referencing Tables 1 and 2. For a full and up-to-date list of the keywords we are tracking, please see the “keywords.txt” file in the GitHub repository (a list of the accounts we are tracking can be found in the “accounts.txt” file) [24]. Some of the keywords may appear earlier than the initial listed track date in Table 1, as we systematically ran the same keywords through Twitter's search API to collect past instances of the keywords shortly after adding the keywords to be tracked in real time.

**Table 5:** Breakdown of the most popular languages and the number of associated tweets (v1.8—May 8, 2020).

Language	ISO^a^	Tweets (N=123,113,914), n (%)
English	en	80,698,556 (65.55)
Spanish	es	13,848,449 (11.25)
Indonesian	in	4,196,591 (3.41)
French	fr	3,762,601 (3.06)
Portuguese	pt	3,451,196 (2.80)
Japanese	ja	2,897,046 (2.35)
Thai	th	2,754,627 (2.24)
(undefined)	und	2,711,649 (2.20)
Italian	it	1,615,916 (1.31)
Turkish	tr	1,308,989 (1.06)

^a^ISO: International Organization for Standardization.

#### General Release Notes

In order to use any Twitter-facing libraries, including hydration software, users must first apply for a Twitter developer account and obtain the necessary authentication tokens [28].

The GitHub community has also generously contributed scripts to enable researchers to hydrate the Tweet IDs using *Twarc* [26].

## Discussion

### Overview

We present an initial analysis of our collected data set that verifies that Twitter discourse statistics reflect major events at the time, and leverage Business Insider [29], NBC [30], and CNN [31] released timelines to identify these events of interest during the development of the COVID-19 pandemic. In some of these analyses, there is a dip on March 2, 2020—this was due to internet connectivity failures throughout that specific day. Our discussion is based on analysis done on tweets from release v1.2 (January 21, 2020 to March 31, 2020), while the most recent release is v1.8.

### Hashtags

**Figure 1:**
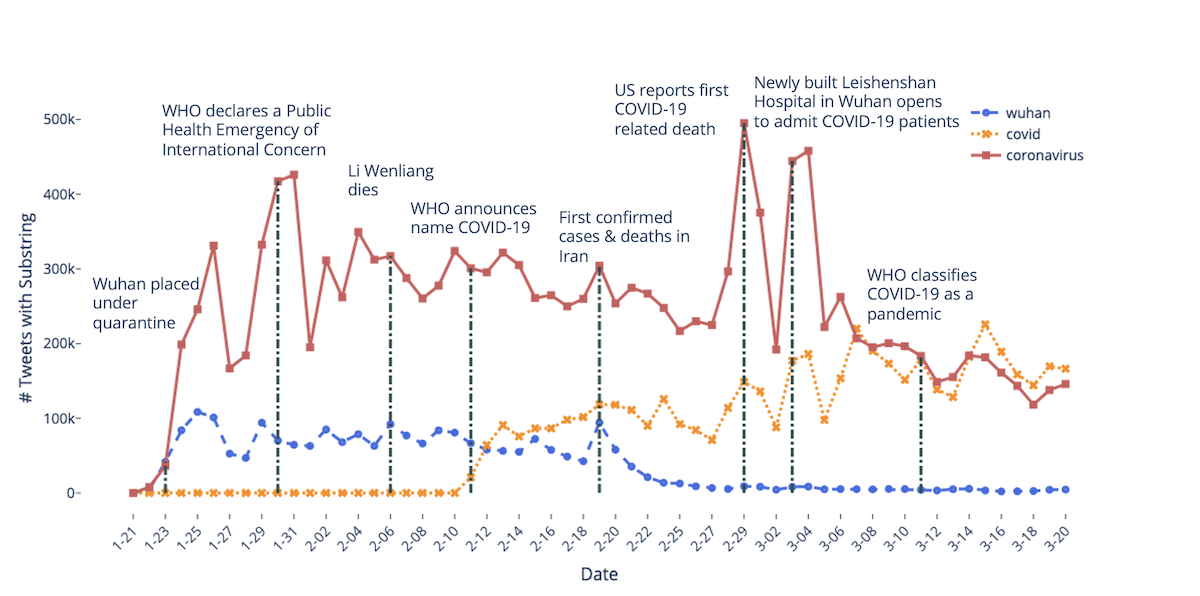
Usage of hashtags containing the substrings “wuhan,” “covid,” and “coronavirus” over time. COVID-19: coronavirus disease; WHO: World Health Organization.

We tracked the frequency of COVID-19-related hashtags, specifically those that contain the substrings “wuhan,” “coronavirus,” and “covid” throughout our collection period (Figure 1). We can see that while hashtags with the substring “coronavirus” consistently remain a more heavily used hashtag in our data set, the hashtag usage spiked on the day the WHO declared COVID-19 a global public health emergency; it also spiked on the day the United States announced the first COVID-19-related death [2]. We also did not see hashtags referencing “covid” being used until February 11, 2020, when the WHO announced “COVID-19” as the official name for the novel coronavirus disease. The keyword “wuhan” in hashtags experienced consistent usage until late February, then steadily declined, which reflects the decrease in cases in China and the global spread of the virus.

### Languages

**Figure 2:**
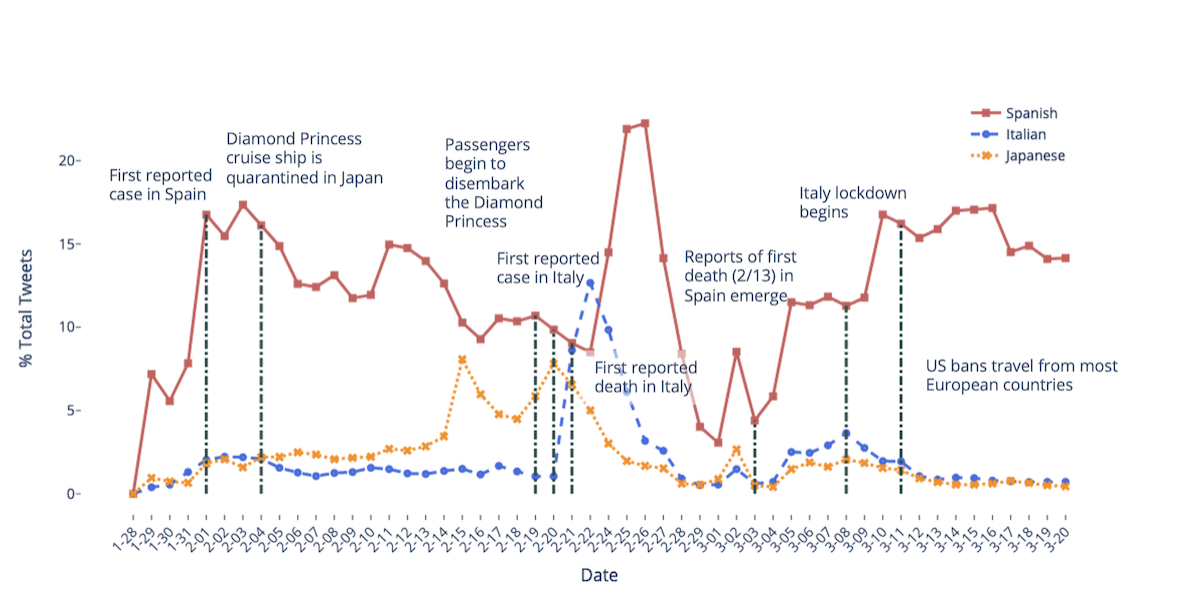
Tweets in Spanish, Italian, and Japanese over time (our multilingual database began data collection after January 28, 2020).

We then examined the percentage of total tweets posted in different languages (Figure 2). Although English is the most prominent language in our data set, we excluded English from this analysis to better visualize tweet activity in countries that experienced COVID-19 outbreaks earlier in the timeline. In particular, we found that Japanese tweet activity increased steadily after the cruise ship Diamond Princess was quarantined off the coast of Yokohama, Japan, with a peak around the time when passengers began to disembark [32].

There was also a significant spike in tweets from Italy when the first case related to COVID-19 was reported in Lodi, Italy, and first death was seen in Veneto [33]. We also observed a peak in the percentage of Spanish tweets after the first COVID-19 case in Spain was announced on February 1, 2020 [34] and a steady increase in the percentage of Spanish tweets after reports of the first COVID-19-related death began to emerge (the death itself occurred on February 13th, but the cause was diagnosed postmortem) [35].

### Verified Users

**Figure 3:**
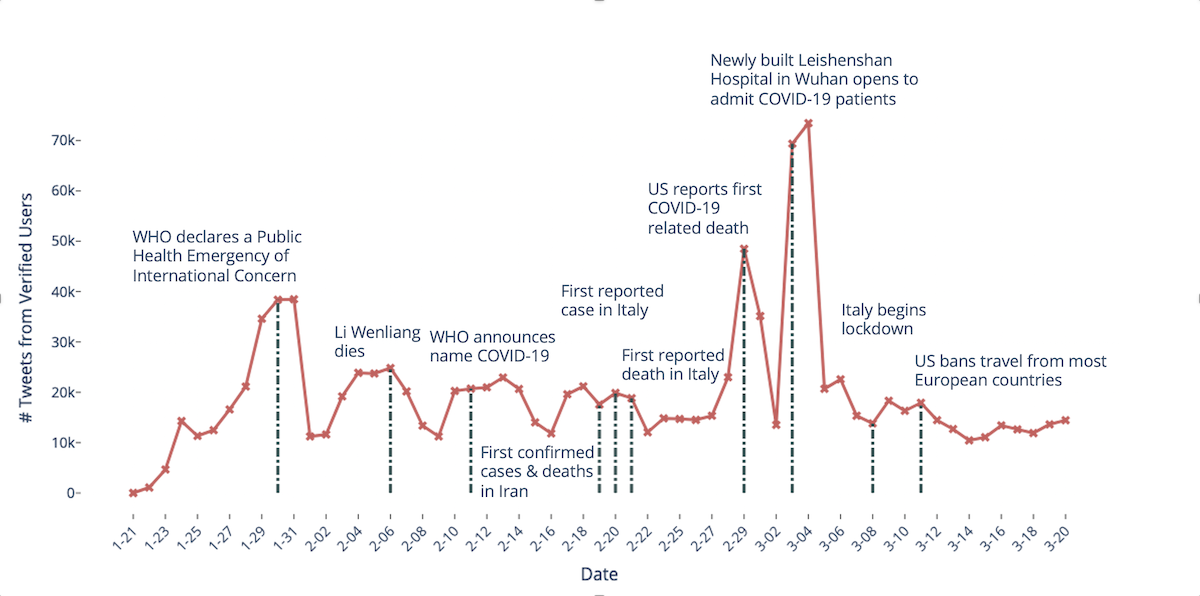
Number of tweets from verified users over time. COVID-19: coronavirus disease; WHO: World Health Organization.

Verified users on Twitter have been identified by Twitter as accounts of public interest and are verified to be authentic accounts [36]. We observed that the verified accounts, which include news sources and political figures, are the most active when major events occur, as seen in Figure 3. This is to be expected since influential figures and news sources often weigh in and report on breaking news in real time using Twitter as a platform to amplify their messaging. As the United States also drives much of the discourse on Twitter, it is therefore unsurprising that there is a major spike in activity from verified users when the country experienced its first COVID-19-related death.

### Limitations

There are several limitations to our data set. We collect our data set leveraging Twitter’s free streaming API, which only returns 1% of the total Twitter volume, and the volume of tweets we collected continues to be dependent on our filter endpoint and network connection [37].

While our data set is a multilingual data set, containing tweets in over 67 languages, the keywords and accounts we have been tracking and continue to track have been mostly English keywords and accounts. Thus, there is a significant bias in favor of English tweets in our data set over tweets in other languages.

Despite these limitations, our data collection gathers over 1 million tweets a day from the 1% of tweets available to us through Twitter’s API, and our data set contains on average 35% non-English tweets. Our collection begins in late January, capturing tweets during many major developments, and we plan on continuing collecting tweets for the foreseeable future.

## Abbreviations

API: application programming interface
COVID-19: coronavirus disease
IRB: institutional review board
WHO: World Health Organization
